# Formulation Development of Solid Self-Nanoemulsifying Drug Delivery Systems of Quetiapine Fumarate via Hot-Melt Extrusion Technology: Optimization Using Central Composite Design

**DOI:** 10.3390/pharmaceutics16030324

**Published:** 2024-02-26

**Authors:** Prateek Uttreja, Ahmed Adel Ali Youssef, Indrajeet Karnik, Kavish Sanil, Nagarjuna Narala, Honghe Wang, Rasha M. Elkanayati, Sateesh Kumar Vemula, Michael A. Repka

**Affiliations:** 1 Department of Pharmaceutics and Drug Delivery, School of Pharmacy, The University of Mississippi, University, MS 38677, USA; puttreja@go.olemiss.edu (P.U.); aayousse@go.olemiss.edu (A.A.A.Y.); irkarnik@go.olemiss.edu (I.K.); ksanil@go.olemiss.edu (K.S.); nnarala@go.olemiss.edu (N.N.); hwang8@go.olemiss.edu (H.W.); rmelkana@go.olemiss.edu (R.M.E.); svemula@olemiss.edu (S.K.V.); 2 Department of Pharmaceutical Technology, Faculty of Pharmacy, Kafrelsheikh University, Kafrelsheikh 33516, Egypt; 3 Pii Center for Pharmaceutical Technology, The University of Mississippi, University, MS 38677, USA

**Keywords:** quetiapine fumarate, hot-melt extrusion, self-nanoemulsifying drug delivery systems, central composite design

## Abstract

Quetiapine fumarate (QTF) was approved for the treatment of schizophrenia and acute manic episodes. QTF can also be used as an adjunctive treatment for major depressive disorders. QTF oral bioavailability is limited due to its poor aqueous solubility and pre-systemic metabolism. The objective of the current investigation was the formulation development and manufacturing of solid self-nanoemulsifying drug delivery system (S-SNEDDS) formulation through a single-step continuous hot-melt extrusion (HME) process to address these drawbacks. In this study, Capmul^®^ MCM, Gelucire^®^ 48/16, and propylene glycol were selected as oil, surfactant, and co-surfactant, respectively, for the preparation of S-SNEDDS. Soluplus^®^ and Klucel™ EF (1:1) were selected as the solid carrier. Response surface methodology in the form of central composite design (CCD) was utilized in the current experimental design to develop the S-SNEDDS formulations via a continuous HME technology. The developed formulations were evaluated for self-emulsifying properties, particle size distribution, thermal behavior, crystallinity, morphology, physicochemical incompatibility, accelerated stability, and in vitro drug release studies. The globule size and emulsification time of the optimized SNEDDS formulation was 92.27 ± 3.4 nm and 3.4 ± 3.38 min. The differential scanning calorimetry (DSC) and powder X-ray diffraction (PXRD) studies revealed the amorphous nature of the drug within the formulation. There were no drug-excipient incompatibilities observed following the Fourier transform infrared (FTIR) spectroscopy. The optimized formulation showed an extended-release profile for 24 h. The optimized formulation was stable for three months (last time-point tested) at 40 °C/75% RH. Therefore, the developed S-SNEDDS formulation could be an effective oral delivery platform for QTF and could lead to better therapeutic outcomes.

## 1. Introduction

Oral dosage forms account for approximately 80% of the pharmaceutical market due to their ease of use, low production costs, non-sterile production, ability to be administered by the patient and the fact that high drug loading can be achieved easily [1,2]. However, poor aqueous solubility is the primary formulation development drawback of around 40% of currently available oral drugs and new chemical entities. It is estimated that about 75% of the drugs that are under development have low water solubility [3]. Classes II (low solubility and high permeability) and IV (low solubility and low permeability) in the Biopharmaceutical Classification System (BCS) are the two classes that include all poorly water-soluble drugs [4]. Many formulation strategies have emerged in recent decades to improve the oral bioavailability of poorly water-soluble drugs by increasing their apparent solubility in gastrointestinal fluids [5]. Among these methods, lipid-based formulations cover a broad spectrum of drug delivery systems that offer several benefits when administered orally, including increased drug-apparent solubility, improved permeability and decreased pre-systemic metabolism [6].

Self-nanoemulsifying drug delivery systems (SNEDDS) are a promising formulation development strategy for increasing the aqueous solubility of hydrophobic drugs. These multicomponent systems include an oil or lipid, a surfactant or a mixture of surfactants, and, optionally, a co-solvent [7,8,9]. The globule size of SNEDDS is typically within the range of 10–100 nm [10]. When SNEDDS encounter digestive fluids and gastrointestinal motility, they spontaneously convert into nanoemulsions that can solubilize the formulated drug [11]. However, most research endeavors related to SNEDDS are focused on liquid SNEDDS (L-SNEDDS). Although L-SNEDDS can be prepared very fast with rudimentary methods along with a high drug loading capacity, there are several drawbacks associated with these liquid formulations [8,12]. For example, Neoral^®^ and Fortovase^®^, are soft gelatin capsules, prepared with time- and resource-intensive manufacturing techniques with a high possibility for formulation leakage outside the capsule shell. In addition, there are a few products with low market potential because of numerous obstacles such as high production costs, poor stability and mobility, the potential for drug precipitation upon dilution, the dearth of predictive in vitro methods, and the need for sophisticated manufacturing equipment [13].

Hence, novel solid dosage forms that retain the benefits of the L-SNEDDS formulation while minimizing its drawbacks are required. It is possible to convert L-SNEDDS into solid SNEDDS (S-SNEDDS) using several well-described production techniques, including adsorption to a solid carrier, wet granulation, spray drying, freeze drying, and supercritical fluid processes. The adsorption of L-SNEDDS into a solid carrier is the most widely employed formulation method for preparing S-SNEDDS at present [14]. S-SEDDS provides many benefits, including enhanced permeability, controlled drug release, and a longer GI residence time. Moreover, the production cost is minimal and the physicochemical stability is further improved [15,16]. Recently, hot-melt extrusion (HME) technology has emerged as a viable method for manufacturing S-SNEDDS. HME is the primary technology for preparing amorphous solid dispersions (ASDs) [17,18] and has also been explored in the manufacturing of pharmaceutical co-crystals [19,20], polymeric implants [21], pellets [22], and lipid nanoparticles [23]. Because the process does not require solvents and is easily scalable for continuous manufacturing, HME is an intriguing option for the advancing S-SNEDDS. In addition, HME requires less time investment than many other primitive technologies. To date, limited investigations have been reported to prepare S-SNEDDS using HME [14].

One of the recent compounds used in the treatment of schizophrenia and bipolar disorders is quetiapine, which is commercialized as a fumarate salt (QTF). The atypical antipsychotic QTF is a dibenzo thiazepine derivative that was approved by the Food and Drug Administration (FDA) in 1997. QTF is classified as a class II drug under the BCS. QTF’s poor solubility and significant hepatic first-pass metabolism contributed to its low oral bioavailability (9%) [24,25].

The current research focuses on continuously manufacturing the S-SNEDDS of QTF using HME technology for the first time. The developed systems could improve drug solubility, promote lymphatic transport to reduce first-pass metabolism, and extend QTF release following oral administration. The effect of the formulation variables on the performance of developed S-SNEDDS was investigated using a central composite design (CCD). The developed systems were optimized based on globule size and emulsification time. The optimized formulation was evaluated for in vitro release, drug-excipient incompatibility, thermal behavior, and stability studies.

## 2. Materials and Methods

### 2.1. Materials

QTF was purchased from SPECTRUM^®^ CHEMICAL NFG CORP (New Brunswick, NJ, USA). The lipid excipients Gelucire 48/16, and Gelucire 44/14 were kindly gifted by Gattefossé (Paramus, NJ, USA). Hydroxypropyl cellulose (Klucel™ EF; HPC-EF) was gifted by Ashland Global Chemicals Company (Burlington, NJ, USA). Polyvinyl caprolactam–polyvinyl acetate–polyethylene glycol graft copolymer (Soluplus^®^) was received as a generous gift from BASF Chemical Co. (Ludwigshafen, Germany). Capmul MCM (Medium Chain mono and di-glycerides) was gifted by ABITEC Corporation (Columbus, OH, USA). Propylene glycol and other excipients used for screening studies were purchased from Sigma-Aldrich (St. Louis, MO, USA). All other chemicals and solvents utilized in the current investigation were of analytical grade and were purchased from Fischer Scientific (St. Louis, MO, USA). The size 00 hard gelatin capsules were procured from Total Pharmacy Supply Inc. (Arlington, TX, USA).

### 2.2. Analytical Method

QTF quantification was achieved based on the HPLC method described under the United States Pharmacopeia (USP) monograph of Quetiapine tablets. QTF was quantified using a Waters HPLC-UV system (Waters Corp., Milford, MA, USA) with a UV/VIS detector. The HPLC analysis was achieved on a Phenomenex Luna^®^ (25 cm × 4.6 mm, 5.0 μm) C18 column. The detection wavelength (λmax) was set to 225 nm during the analysis. The buffer part was prepared by dissolving monobasic potassium phosphate (1.4 g) in water (1000 mL). Then, triethylamine (1 mL) was added, and the pH was adjusted with dilute phosphoric acid to 6.5. The mobile phase was prepared by mixing acetonitrile and the buffer into a 35:65 *v*/*v* ratio. The mobile phase was pumped automatically at a flow rate of 1.2 mL/min through the system. The injection volume was set to 20 μL. The stock solution of QTF was prepared by dissolving the drug in the mobile phase. The samples were processed on the Waters Empower software (https://www.waters.com/nextgen/us/en/products/informatics-and-software/chromatography-software/empower-software-solutions.html) chromatography data system. The calibration curve was linear over the QTF concentration range of 1.0–100 μg/mL.

### 2.3. Screening of Formulation Excipients

The solubility of QTF was screened in various lipids and surfactants by individually adding 10 mg of QTF to 1000 mg of the excipient in glass vials (3 mL). Then, the drug–excipient mixtures were heated at 80 ± 2 °C under continuous magnetic stirring (1000 rpm). The mixtures were then cooled to room temperature and examined visually for any suspended or precipitated drug particles. The excipients that showed no suspended or precipitated drug particles were chosen for the formulation preparation.

### 2.4. Saturation Solubility in Liquid Excipients

The solubility of QTF in the liquid excipients, identified during the screening studies, was evaluated. Briefly, excess QTF was transferred to glass scintillation vials containing the liquid excipient (2 mL). The vials were loaded into a reciprocating water bath (Precision^TM^, Thermo Scientific^TM^, Waltham, MA, USA) set to 25 ± 0.5 °C and operated at 100 rpm for 48 h. The drug–excipient mixtures were centrifuged (AccuSpin 17R centrifuge, ThermoFisher Scientific, Hanover, IL, USA) for 30 min at 13,000 rpm. The supernatant was filtered using a 0.22 µm pore size Nylon membrane syringe filter (Millex^®^ Nylon syringe filter, Millipore Sigma, St. Louis, MO, USA). The filtrate was collected and analyzed for QTF following proper dilution, using the HPLC method described above.

### 2.5. Saturation Solubility in Solid/Semisolid Excipients

The solubility of QTF in the solid lipid excipients, identified during the screening studies, was evaluated following a solubility method, developed by Gattefossé [26]. The solubility of QTF was investigated by differential scanning calorimetry (DSC). Samples for this study were prepared by melting the excipient and dispersing an accurately weighed amount of the drug (0, 50, 100, 150, 200, 250, 275, 300, 350, and 400 mg/g) in the lipid under continuous magnetic stirring. These samples were left overnight at 50 °C to equilibrate. Then, aliquots were loaded in aluminum pans (4–6 mg) to solidify at room temperature for 24 h before DSC analysis (TA Instruments DSC, New Castle, DE, USA). Thermal analysis was conducted between 25 and 200 °C with a heating rate of 10 °C/min. The melting enthalpy of all the samples is measured and plotted against the concentration of the samples (*w*/*w*) to determine the saturation solubility. The drug solubility was recorded when there was a change in the slope of the curve fitting and the evolution of melting enthalpy of the solid lipid excipient as a function of the QTF concentration in the mixture.

### 2.6. Preparation of Physical Mixtures

Accurately measured amounts of oil, surfactant, co-surfactant, and solid carriers were mixed using a mortar and pestle to prepare all physical mixtures (PMs). Briefly, the drug was dissolved in the liquid lipid. Then, the surfactants and co-surfactants were added. The mixtures were levigated for 5 min to form a paste. The paste was then adsorbed onto the polymeric solid carriers––Soluplus^®^ and Klucel™ EF (1:1).

### 2.7. Experimental Design

Based on the literature review and evaluation, the independent variables (factors) that can affect the critical quality attributes of SEDDS were selected [27,28]. Response surface methodology in the form of central composite design (CCD) [25] was utilized in the current experimental design with three independent variables to determine the optimal levels for the mean globule size (GS, nm, Y_1_) and emulsification time (ET, min, Y_2_) at 2 levels assigned to the design. The selected factors were the oil concentration (Capmul^®^ MCM, % *w*/*w*, X_1_), surfactant–co-surfactant ratio (Gelucire^®^ 44/14 or Gelucire^®^ 48/16: Propylene glycol, X_2_), and surfactant type (Gelucire^®^ 44/14 or Gelucire^®^ 48/16, X_3_), which were investigated at five different levels: one central point (X_1_: 15% *w*/*w*, X_2_: 3), level +1 (X_1_: 20% *w*/*w* and X_2_: 4.0), level −1 (X_1_: 10% *w*/*w* and X_2_: 2.0), level +α (X_1_: 4.41421% *w*/*w* and X_2_: 22.0711), and level −α (X_1_: 7.92893% *w*/*w* and X_2_: 1.58579). The α value (1.7321) was calculated by the equation ±$\sqrt{k}$ for k = 3 (three independent factors). The details of the CCD used in this investigation are provided in Table 1 and Table 2. The CCD was generated by the Design-Expert^®^ software (StatEase^®^, version 13.0) with a total of 22 runs with 18 different formulations, including 8 factorial points (levels, ±1), 8 axial points (levels, ±α), and 6 replicates at the central point for pure error estimation. All runs were randomized to minimize the effects of variability of the recorded responses due to systematic errors.

### 2.8. Preparation of S-SNEDDS

PMs (10 g) were fed into a HAAKE 6 mm Minilab II extruder (Thermo Fisher Scientific, Karlsruhe, Germany) at a feeding rate of 0.4 g/min using a volumetric feeder at the extruder barrel temperature of 125 °C from the feeding zone to the die section, rotating with a screw speed of 50 rpm. The torque was continuously observed during the extrusion process. The extrudates were collected at the end of the extruder from a spherical die (2.0 mm). After cooling the extrudate to room temperature, the extruded materials were then subjected to dry ice/liquid nitrogen until they became brittle and finally milled using a coffee grinder for 1–2 min. The milled extrudates were passed through a US #25 mesh sieve with (700 µm aperture) and stored in tightly closed glass scintillation vials in a vacuum desiccator at room temperature until further analysis.

### 2.9. Self-Emulsification Test

The self-emulsification time was assessed by a dispersibility test following the method described by Parmar et al., [29]. SNEDDSs (250 mg) were transferred to a dissolution vessel containing 250 mL of 0.1 N HCl. USP Type II dissolution apparatus with a rotating speed of 50 rpm and a temperature of 37 ± 0.5 °C was used. The time required for the powder to be dispersed and converted into a nanoemulsion was recorded based on visual observations. Moreover, the formed nanoemulsion was visually observed for 24 h for any physical instability issues––phase separation, cracking, creaming, coalescence, or phase inversion. This experiment was conducted in triplicates for each run.

### 2.10. Globule Size (GS), Polydispersity Index (PDI), and Zeta Potential (ZP)

Zetasizer Nano ZS Zen3600 (Malvern Panalytical Inc., Westborough, MA, USA) performed photon correlation spectroscopy at 25 °C in a disposable transparent cell to determine the mean GS and PDI. Using a helium-neon laser, the PDI and GS were measured. The formulations were filtered through a 0.45 µm Nylon filter before measurement. The same instrument was used for a Laser Doppler Velocimetry analysis for ZP measurements. GS, PDI, and ZP were measured in triplicates [30].

### 2.11. Assay and Content Uniformity

Formulations were weighed (250 mg) and transferred to a volumetric flask (100 mL) containing a mixture of methanol and dimethyl sulfoxide into 1:1 *v*/*v*. The extract was vortexed for 5 min at 2000 rpm using Vortex-Genie^®^ 2 (Scientific Industries, Inc., Bohemia, NY, USA) and sonicated (Bransonic^®^ ultrasonic cleaner, Brookfield, CT, USA) for 10 min. The extract (1 mL) was then centrifuged for 20 min at 13,000 rpm using an AccuSpin 17R centrifuge (Fisher Scientific, Hanover, IL, USA). The supernatant was filtered through a 0.45 μm Nylon syringe filter and diluted 100 times with the same extracting solvent before being analyzed for QTF content using the HPLC method.

The uniformity of dosage units was evaluated following the content uniformity test described under the “Uniformity of Dosage Units” chapter in the USP. Ten empty hard gelatin capsules were filled with powder equivalent to 25 mg of QTF from the optimized formulation (~250 mg). For hard capsules containing less than <25 mg drug or <25% drug load, a content uniformity test should be performed. The capsules of each were assayed individually and quantified for QTF content using the HPLC described above.

### 2.12. Thermal Analysis

DSC analysis studies were conducted to determine the melting point (T_m_) of QTF and the solid lipid and glass transition temperatures (T_g_) of both solid carriers. Before analysis, the instrument was calibrated using the indium and sapphire standards for temperature and heat capacity. Samples (~5 mg) were accurately weighed and sealed in regular aluminum pans. The reference was an empty aluminum pan. The samples were equilibrated for 1 minute at 25 °C and then heated to 200 °C at a heating rate of 10 °C/min under the continuous flow of an inert nitrogen purge (50 mL/min). The DSC instrument analyzed data with Trios^®^ software (https://www.tainstruments.com/trios-software/, New Castle, DE, USA).

### 2.13. Fourier Transform Infrared (FTIR) Spectroscopy

The compatibility of the drug and formulation excipients, as well as any interactions between the components of the formulation, were analyzed using an Agilent Cary 660 FTIR spectrometer (Agilent Technologies, Santa Clara, CA, USA). A MIRacle high-pressure clamp was used to compress the sample amount of approximately 10 mg placed on top of the diamond crystal. The specimens underwent 32 scans with a resolution of 8 cm^−1^, spanning from 600 to 4000 cm^−1^. The instrument was installed using a single-bounce, diamond-coated ZnSe internal reflection element, and attenuated total reflection (Pike Technologies, Madison, WI, USA).

### 2.14. Powder X-ray Diffraction (PXRD) Analysis

The solid-state properties of pure formulation components (drug, lipid, and solid carriers) along with the optimized formulation were investigated using the Rigaku X-ray system (D/MAX-2500PC, Rigaku Corp, Tokyo, Japan) using Cu rays (*λ* = 1.54056 Å) with a current of 40 mA and voltage of 40 kV at ambient room temperature. All the scans were performed from 2 to 50° at a rate of 10°/min and width of 0.01°/s.

### 2.15. Scanning Electron Microscopy (SEM)

A JSM-7200FLV scanning electron microscope (JEOL, Peabody, MA, USA) equipped with a 10 kV accelerating voltage was used to investigate the surface structure of the optimized formulations. The samples were affixed to SEM stubs utilizing double-adhesive tape. The preparation of SEM samples involved the placement of 10 mg of the optimized formulation onto a silicon wafer chip, followed by air-drying at room temperature. To prepare the samples for imaging, a fully automated Denton Desk V TSC Sputter Coater (Denton Vacuum, Moorestown, NJ, USA) was used to sputter-coat them with platinum in an argon atmosphere.

### 2.16. Dissolution Studies

The in vitro drug release profiles of the optimized formulation and pure QTF were obtained using SR8-plus Hanson dissolution USP type I apparatus (Hanson SR8-plus™; Hanson Research, Chatsworth, CA, 173 USA). The capsules were added to the dissolution vessels containing 0.1 N HCl (500 mL) [31]. The temperature of the dissolution medium was maintained at 37 ± 0.5 °C, and the rotation speed was set to 50 rpm. Samples were collected at predetermined time intervals (0.5, 1, 2, 4, 6, 8, 12, and 24 h) using a 0.45 m Nylon syringe filter and analyzed using the HPLC instrument. The release profiles were fitted to four mathematical models using DDSolver software, a free add-in program for Microsoft Excel (Office365, 2019, USA), to explore the possible release mechanism.

### 2.17. Stability Studies

The optimized formulation was investigated for physicochemical stability upon storage under accelerated (40 ± 2 °C/75 ± 5% RH) storage conditions for 3 months. The optimized formulation was evaluated for any change in the GS, PDI, ZP, emulsification time, QTF content, and dissolution profile upon storage.

### 2.18. Statistical Analysis

Design-Expert^®^ software (StatEase^®^ Inc., Minneapolis, MN, USA, version 13.0) was used in this study for formulation optimization and statistical evaluation. The differences were considered statistically significant when the *p*-value was <0.05.

## 3. Results and Discussions

### 3.1. Screening Studies

Selecting a suitable oil, surfactant, or co-surfactant is essential in developing SNEDDS formulations. A single excipient within the formulation matrix might not be able to dissolve the entire drug load. However, combining two or more compatible excipients could help accommodate more drug molecules, thus solubilizing the drug molecules within the formulation matrix without precipitation or recrystallization [22]. The solubility of QTF was screened in oil, surfactant, and co-surfactant excipients as shown in Table 3. Based on visual observations, QTF dissolved in Capmul^®^ MCM, Gelucire^®^ 44/14, Gelucire^®^ 48/16, and propylene glycol, and Transcutol^®^ HP did not show precipitation after cooling the drug–excipient mixture to room temperature. Therefore, these excipients were selected for further evaluation.

### 3.2. Saturation Solubility of Liquid Excipients

**Figure 1:**
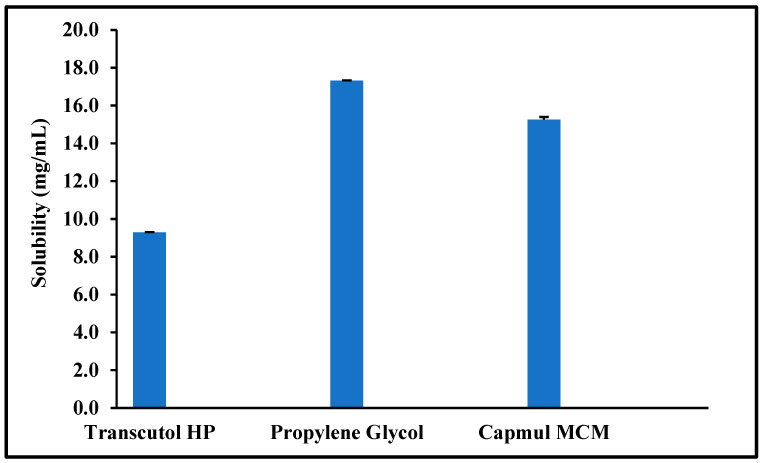
Saturation solubility of quetiapine fumarate (QTF) in the selected liquid excipients (Mean ± SD, n = 3).

Saturation solubility studies were carried out for the selected formulation excipients to maximize the drug load in the selected lead liquid excipients. Moreover, these studies helped compare the three successful surfactants: Transcutol^®^ HP, Gelucire^®^ 50/13, and Gelucire^®^ 44/14. The saturation solubility results were in the following order for liquid excipients, as shown in Figure 1: propylene glycol > Capmul^®^ MCM > Transcutol^®^ HP.

### 3.3. Saturation Solubility in Solid Excipients

**Figure 2:**
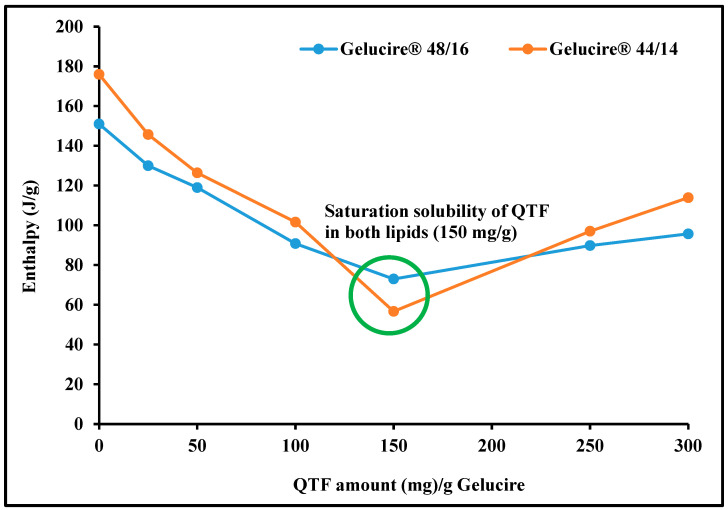
Saturation solubility of quetiapine fumarate in solid excipients (Gelucire^®^ 48/16 and Gelucire^®^ 50/13) by differential scanning calorimetry.

The saturation solubility of QTF in Gelucire^®^ 48/16 and Gelucire^®^ 44/14 is depicted in Figure 2. As stated by Raoult’s law, the change of melting enthalpy is linked to the concentration of the drug dissolved within the excipient [26]. Therefore, the main principle in calculating the saturation solubility in this study is that increasing drug concentration decreases the melting enthalpy of the solid solvent until the point of saturation solubility is reached [32]. Figure 2 shows that the melting enthalpy of the solid solvent increases above the point of saturation solubility due to the increase in the energy required to melt the solid solvent. After the saturation solubility point, the melting enthalpy of the solid solvent started to increase again. Moreover, both figures of the investigated surfactants showed a similar trend in the melting enthalpy of solid solvent against concentration with a saturation solubility of 150 mg of QTF in 850 mg Gelucire (1:5.7 ratio).

Among the three investigated surfactants, Gelucire^®^ 48/16 and Gelucire^®^ 44/14 showed higher drug solubility than Transcutol^®^ HP, which can be attributed to its good solubilizing potential. Gelucire^®^ is a water-soluble non-ionic surfactant suitable for solubilizing lipophilic drugs because it can form a micellar system when formulated as a binary mixture with the drug alone or in the presence of any co-surfactant [32]. Generally, surfactants with a high HLB value like Gelucire^®^ are preferred for developing SEDDSs. Higher HLB values help improve hydrophilicity, thus resulting in the rapid formation of o/w emulsion due to the rapid spreading of the formulation when it meets the dispersion medium. In addition, Gelucire^®^ can adsorb to the surface of the oil globules, preventing the coalescence of oil globules and helping in the stability of the formed droplets for a longer time until absorption. Therefore, Gelucire^®^ was chosen as the primary surfactant in this investigation.

### 3.4. Formulation Development

**Figure 3:**
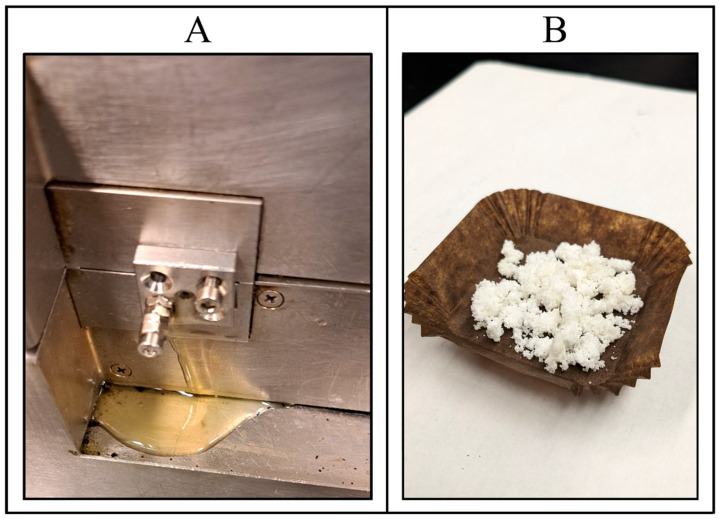
(**A**) Hot melt extrusion failure with oil (Capmul^®^ MCM) concentration above 20% *w*/*w* showing liquid extrudate after cooling to room temperature; and (**B**) quetiapine fumarate-loaded HME S-SNEDDS.

The HME preparation process for QTF-loaded S-SNEDDS required a single step of continuous extrusion: feeding the blend into the extruder and collecting the extrudate from the discharge zone. Another step of size reduction followed this step. However, many preliminary tests were performed to determine the ranges of extruder operating conditions and the elemental composition of the formulation to guarantee an adequate extrusion process as well as the ability of the extrudates to form a suitable emulsion after fast emulsification process in an acidic medium. The process temperature used for preliminary tests was 100–140 °C based on the melting of the lipid excipients and solid carriers. The melting point of QTF (177 °C) was not considered because the drug was completely dissolving in the liquid lipid before the extrusion process. The use of oil concentration above 20% *w*/*w* with all tested solid carriers gave a liquid extrudate which was not solidifying to a solid mass that was difficult to give a powder during the size reduction process as shown in Figure 3.

The initial attempts during extruding S-SNEDDS of QTF using Neusilin^®^ US2 and Kollidon VA^®^ 64, along with the lipid excipients, were unsuccessful because very long emulsification times were observed (>20 min). The addition of the solid plasticizer HPC and hydrophilic adsorbent Soluplus^®^, which was adjusted to a mass ratio of 1:1, also made the successful extrusion of the formulation using up to 20% *w*/*w* oil concentration. Based on these preliminary findings, the formulations were processed at a screw speed of 75 rpm and a processing temperature of 125 °C. It was assumed, that at these processing parameters, QTF and the lipid excipients could have interacted in a molten state and simultaneously distributed the molten components onto the solid carriers (HPC: Soluplus^®^, 1:1), which can be confirmed using solid-state characterization tests, namely DSC and PXRD. The residence time of the extrusion process was around 3 min for all S-SNEDDS formulations. The low torque (11–12%) values observed during the extrusion process suggest that the process was facilitated by the lubrication provided by the molten component and the appropriate balance between the proportion of molten material and solid carrier. The material obtained from the discharge zone exhibited the characteristics of a solid waxy substance. The waxy substance was transformed into a solid state after undergoing a congealing process for 10 min at room temperature. Cryogenic milling was employed to mill the solid material. The milled product underwent encapsulation into gelatin capsules and was eventually stored in a high-density polyethylene (HDPE) bottle for follow-up analysis and investigation. Emulsification time [17], surfactant concentration [15], and oil concentration [15] are considered to be the key factors in S-SNEDDS preparation because they can affect emulsification time [17] and globule size [15]; therefore, they were selected for the central-composite design as the independent factors.

### 3.5. Statistical Analysis of the Applied CCD

CCD was used to investigate the main and interaction effects of independent variables, selected based on literature review, on GS (nm), and ET (min). The experimental runs (randomized) with the actual composition of each variable in every single run and the corresponding responses obtained for GS (nm) and ET (min) are shown in Table 4.

#### 3.5.1. Effect of Independent Factors on GS (Y_1_)

The least-square second-order polynomial model equation that describes the relationship between the independent variables and the mean GS obtained from CCD at a 95% confidence level is given below:

Y_1_ = 143.87 + 58.08 X_1_ − 20.53 X_2_ + 23.23 X_3_ − 5.31 X_1_X_2_ − 0.7507 X_1_X_3_ − 3.9 X_2_X_3_ + 15.34 X_1_^2^ + 16.52 X_2_^2^ − 3.46X_1_X_2_X_3_ − 8.79 X_1_^2^ X_3_ − 1.81 X_2_^2^ X_3_ (1)

The model F-value (1011.38) obtained from ANOVA testing (Table 5) implies the model for GS is significant with only a 0.01% chance that the large F-value could occur due to noise. The observed lack-of-fit was not significant (F-value = 3.98, *p*-value = 0.1010) with a 10.10% chance that a lack-of-fit F-value this large could occur due to noise. Furthermore, the value of the predicted regression coefficient (R^2^ = 0.9942) was in reasonable agreement with the adjusted regression coefficient (R^2^ = 0.9981) because the difference is less than 0.2, which implies that the predicted GS values from the model are within a 95% confidence interval (CI) of the observed/experimental values. Out of the 22 experimental runs suggested by the software, the smallest GS (87.1 ± 4.8 nm) was observed for the ninth run, while the largest GS (256.7 ± 0.9 nm) was observed for the seventeenth run (Table 4).

**Figure 4:**
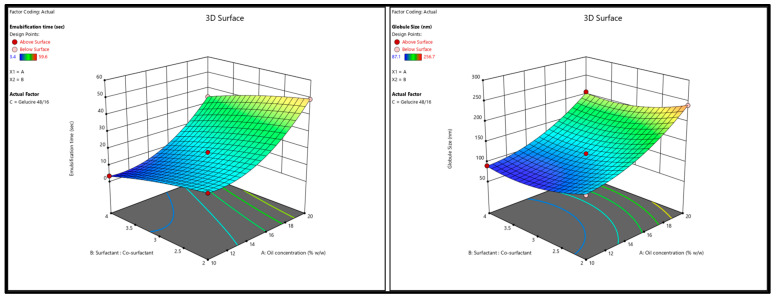
Response surface 3D plots showing (**A**) the effect of oil concentration and surfactant–co-surfactant on the globule size and (**B**) emulsification time of QTF S-SEDDS.

A positive sign before a variable (X_1_; oil conc.) in the polynomial equations signifies that the response (GS) increases with the increasing level of the factor, while a negative sign (X_2_; surfactant–co-surfactant ratio) indicates that the response and the factor have a reciprocal relationship—the response will decrease with an increase in the level of the variable. As shown in Figure 4A and Equation (1), SNEDDS GS can increase as the oil concentration rises; this is likely because the insufficient surfactant cannot effectively cover the oil globules at the higher oil concentration, leading to coalescence [33]. Furthermore, an increase in the surfactant concentration can result in smaller globule sizes; this may be because surfactant molecules tend to absorb the oil–water interface, where they can stabilize the oil globules and prevent coalescence. On the other hand, inadequate amounts of surfactant molecules at low concentrations may result in larger oil globules and less stable emulsions because they cannot form a stable interfacial layer around each droplet. The globule size can be decreased and emulsion stability maintained by increasing the surfactant concentration, which results in a more stable interfacial layer [34].

#### 3.5.2. Effect of Independent Factors on ET (Y_2_)

ET is an essential parameter for characterizing S-SNEDDS. ET can impact the characteristics and performance of the resulting S-SNEDDS. Factors such as the oil concentration and surfactant–co-surfactant play a vital role in attaining optimal ET. The least-square second-order polynomial model equation that describes the relationship between the independent variables and the mean ET obtained from CCD at a 95% confidence level is given below:

Y_2_ = 18.55 + 7.55 X_1_ − 7.14 X_2_ + 0.8833 X_3_ + 0.4625 X_1_X_2_ − 8.27 X_1_X_3_ + 0.0602 X_2_X_3_ + 5.59 X_1_^2^ + 1.28 X_2_^2^ + 0.7125 X_1_X_2_X_3_ − 4.1 X_1_^2^ X_3_ + 2.64 X_2_^2^ X_3_ (2)

The results from ANOVA testing (Table 5) showed that the model F value (1928.94) of the selected quadratic model for the ET indicated a significant model with only a 0.01% chance that this F-value could occur due to noise. In addition, the observed lack-of-fit was insignificant (F-value = 4.57, *p*-value = 0.0815) with an 8.15% chance that the lack of fit F-value this large could occur due to noise. Furthermore, the value of regression coefficients, the adjusted and predicted R^2^ for the regression model was 0.9990 and 0.9969, respectively, which indicates that the model predicted values within 95% CI of the practical ET values. It is worth mentioning that adequate precision for both responses (signal-to-noise ratio)—102.8893 for Y_1_ and 185.8618 for Y_2_—was more significant than 4 (desirable) which indicates an adequate signal and, thus, the selected model can be used to navigate the design space. ET obtained from the 22 experimental runs ranged from 3.4 ± 0.05 min (run# 17) to 59.6 ± 0.7 min (run# 11) as given in Table 4. As shown in Figure 4B and Equation (2), the increase in oil concentration had a positive effect on the ET of QTF-loaded S-SNEDDS which could be due to the simultaneous increase in viscosity of the formulation and hence the prolonged emulsification time [35]. In contrast, the higher the percentage of the surfactant mixture system, the greater the spontaneity of emulsification which is due to the easy diffusion of the aqueous phase to the oil phase, resulting in significant interfacial disruption and discharge of the oil droplets into the bulk aqueous phase [36].

#### 3.5.3. Optimization and Validation

Formulation development heavily relies on the emulsion’s GS because it affects its physical stability, chemical stability, viscosity, in vivo efficacy, and toxicity. Emulsions with the smallest size tend to be the most physically stable, so it is best to develop a formulation with a minimal GS and narrow size distribution. The lipase metabolism of lipids, as well as their interactions with plasma proteins and absorption by the mononuclear phagocyte system (MPS), are profoundly affected by the GS of the lipid phase of emulsions in vivo. Emulsions with GS > 200 nm cannot reach tissues including bone marrow and the small intestine, which are poorly supplied with MPS cells. This outcome suggests that the MPS plays a more significant influence in their clearance than the lipoprotein lipases (LPL). Due to their increased surface area, emulsions with a GS that is less than 100 nm are more vulnerable to LPL, hepatic (HL), or pancreatic (PL) lipase activity. Droplets of lipid emulsions less than 400 nm in diameter are concentrated in solid tumors, peripheral tissues, and inflammatory zones [37]. On the other hand, ET is a crucial index for determining how effectively emulsions are formed. When SEDDSs are diluted in water while gently stirring, they should disperse quickly and thoroughly [27].

**Figure 5:**
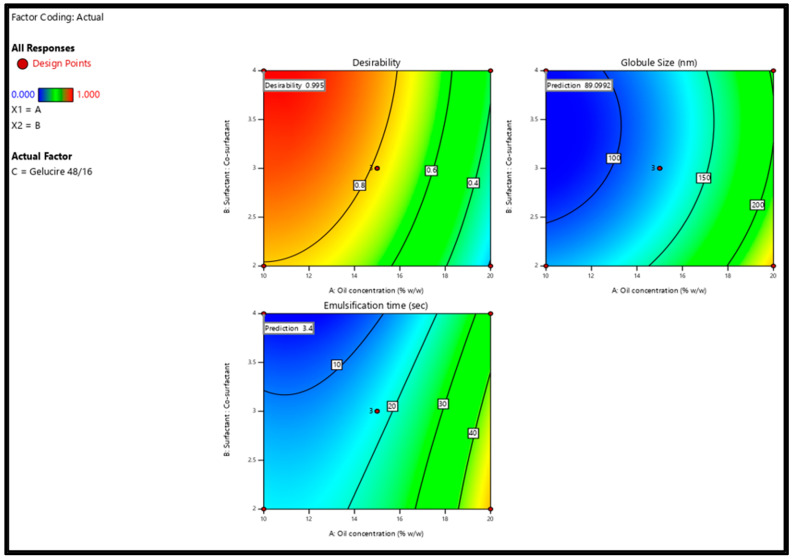
Two-dimensional contour plots show the desirability and predicted response for the proposed solution.

After analyzing responses and building up good regression models, the optimization step was performed to select the level of the factors. The goal for the responses was set to minimize GS (nm) and ET (min). The criteria of the variables and responses for the optimization step are shown in Table 6. In order to achieve the desired responses with 95% confidence intervals (CIs), the software suggested one solution similar to the composition of the F17 formulation (Table 2). The suggested solution is graphically represented by the two-dimensional contour plots shown in Figure 5. To check the validity of the selected model, a fresh S-SNEDDS formulation (n = 3) was prepared to compare the observed/experimental GS and ET values against the software predicted values as given in Table 7. The mean of the experimental GS and EE values was within 95% CI of the software values.

### 3.6. Assessment of Self-Emulsification

**Figure 6:**
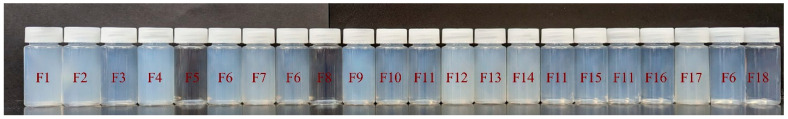
Nanoemulsions obtained after the dispersions of Quetiapine fumarate hot-melt extrudes S-SNEDDS in water.

The assessment test showed that the SNEDDS formulation was homogeneous, transparent, and spontaneously dispersed upon aqueous dilution. Figure 6 represents the appearance of the SNEDDS formulations after aqueous dilution (1 in 1000 *w*/*w* ratio) with water at room temperature (37 ± 0.5 °C). The results for emulsification time are presented in Table 4.

### 3.7. PDI, ZP, and Drug Content

The polydispersity index values of the SNEDDS formulations were less than 0.9 with 0.24 ± 0.03 for the optimized formulation which is a good indication for narrow size distribution after the self-emulsification process. ZP determines the surface charge of dispersed oil droplets and plays a critical role in the physical stability of colloidal dispersions. ZP values ranged from −6.5 ± 2.2 to −23.0 ± 1.8.

Each formulation had QTF content within the acceptance limits of the label’s content (±10%) since the assay in all formulations varied from 91.0 ± 2.3 to 104.9 ± 1.8% (Table 8).

### 3.8. Content Uniformity

The quetiapine fumarate-loaded SNEDDS capsules met the USP acceptance criteria for content uniformity as presented in Table 9.

### 3.9. Differential Scanning Calorimetry

**Figure 7:**
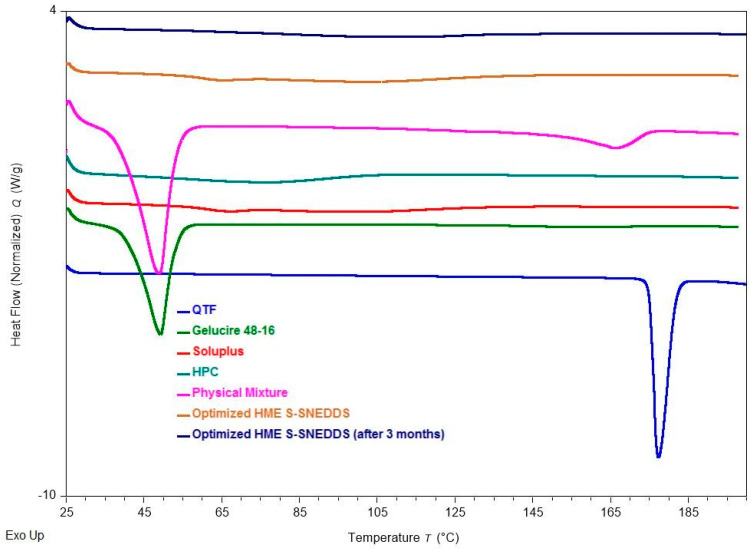
DSC thermograms of quetiapine fumarate, hydroxypropyl cellulose (HPC), Soluplus^®^, Gelucire^®^ 48/16, physical mixture (optimized formulation), optimized HME S-SNEDDS (3-month stability at 40 °C/75 ± 5% RH), and optimized HME S-SEDDS (fresh formulation).

Direct thermal scanning calorimetry (DSC) is a popular thermal analytic tool for detecting both endothermic (melting) and exothermic (crystallization, oxidative breakdown, etc.) transitions [38]. DSC was used to investigate the solid-state properties of the QTF, Gelucire^®^ 48/16, Soluplus^®^, HPC, physical mixture, and extruded optimized formulation (Figure 7). The endothermic melting peak of the pure drug at 177 °C in the thermal scan demonstrates its crystalline form. However, Gelucire^®^ 48/16, a lipid excipient, has melting points that peak around 48 °C. A straight line without endothermic melting peaks was found in the thermal scan of both pure solid carriers (Soluplus^®^, HPC), demonstrating their amorphous nature. The thermogram of the physical mixture for the optimized formulation revealed a small endothermic peak of the drug, because the drug is dissolving in the molten excipient. The drug melting peak did not appear in the thermal scans performed on the extruded optimized formulation. As per the literature, the lack of a drug melting peak during the thermal scan can be explained by either the drug’s amorphous form or its dissolution within the lipid excipient [1,2].

### 3.10. FTIR

**Figure 8:**
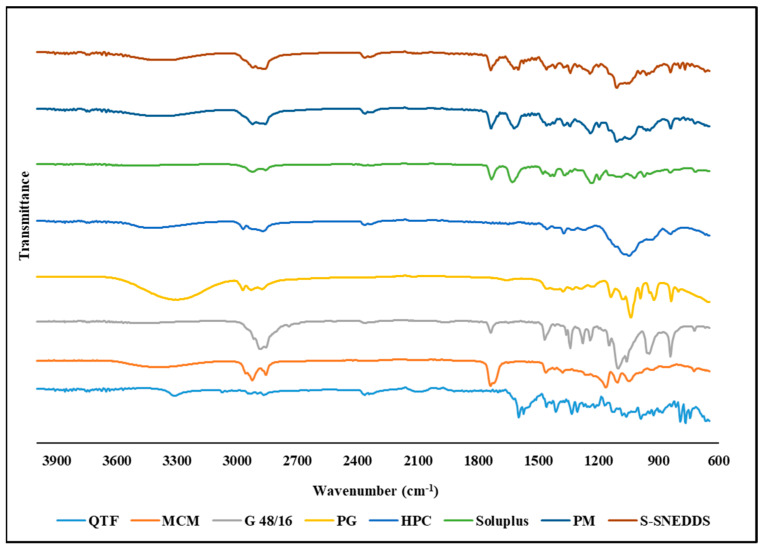
FTIR spectra of quetiapine fumarate (QTF), Capmul^®^ MCM, hydroxypropyl cellulose (HPC), Soluplus^®^, Gelucire^®^ 48/16 (G 48/16), propylene glycol (PG), physical mixture (PM), and optimized HME S-SNEDDS.

FTIR analysis is used to probe the drug–excipient compatibility and the development of interactions between the formulation constituents. The shift or disappearance of the frequency of functional characteristic bands is a reliable indicator of the interactions, which are mostly based on changes at the molecular level [24]. FTIR spectra of QTF, Capmul^®^ MCM, Gelucire^®^ 48/16, propylene glycol, Soluplus^®^, HPC, physical mixture, and S-SENDDS formulation are shown in Figure 8. Pure QTF exhibits characteristic bands at 3749.7 cm^−1^ (O-H stretching), 1599.02 cm^−1^ (C=C stretching), 1457.38 cm^−1^ (C-N bending), and 1334.38 cm^−1^ (C-N stretching). Capmul^®^ MCM showed characteristic bands at 2855.14 cm^−1^ (C–N stretching), 1736.93 cm^−1^ (C=O stretching), and 1107.01 (C–O stretching) while Gelucire^®^ 48/16 showed characteristic bands at 2884.95 cm^−1^ (C–H stretching), 1737.03 cm^−1^ (C=O stretching), 1464.84 cm^1^ (C–H bending), 1341.84 cm^−1^ (S=O stretching) and 842.37 cm^−1^ (C=C bending). FTIR spectrum of propylene glycol showed characteristic bands at 838.65, 991.47, 1039.91, 1136.84, and 2970.68 cm^−1^. Whereas the characterization of Soluplus^®^ has shown characteristic bands at 1733.21 cm^−1^ (C=O stretching), 1628.84 cm^−1^ (C=C stretching), 1233.74 cm^−1^ (C–O stretching), the characteristic bands for HPC were observed at 1370.28 cm^−1^ (O–H bending) and 1047.48 cm^−1^ (CO–O–CO stretching). The physical mixture and the optimized S-SNEDDS formulation showed the same characteristic bands at 2858.86, 1736.93, 1617.66, 1341.84, 1241.2, 1110.74, and 842.37 cm^−1^. In the case of S-SNEDDS, it demonstrated that QTF did not significantly interact with the excipients during the HME process.

### 3.11. Powder XRD

**Figure 9:**
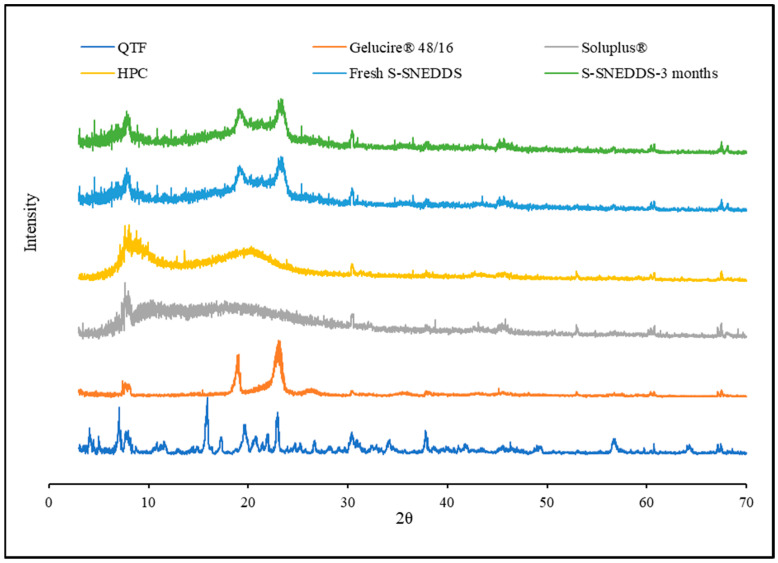
XRD diffractograms of quetiapine fumarate, hydroxypropyl cellulose, Soluplus^®^, Gelucire^®^ 48/16, optimized HME S-SNEDDS (3-month stability at 40 °C/75 ± 5% RH), and optimized HME S-SEDDS (fresh formulation).

Powder X-ray diffraction has been used for qualitative analysis to determine the crystallinity of a material [39]. QTF crystallinity was confirmed by XRD, which revealed peaks at 7.03°, 15.87°, 17.25°,19.64°, and 22.98°. The absence of QTF characteristic peaks in both the optimized and the stability-optimized S-SNEDDS samples shows that the formulation is amorphous and stable (Figure 9). The DSC and PXRD results demonstrate the solubility of QTF in the selected formulation excipients. The amorphous conversion of QTF contributed to the homogeneous distribution of the drug onto the solid carrier.

### 3.12. SEM

**Figure 10:**
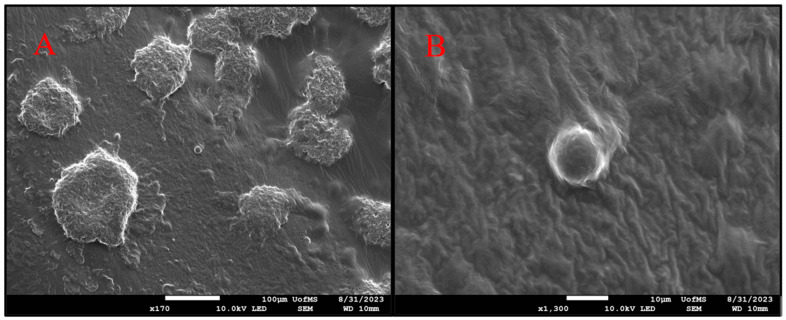
Scanning electron microscopy (SEM) images of dried emulsions of quetiapine fumarate S-SNEDDS following the process of self-emulsification; two different magnifications (**A**) 170× (**B**) 1300×.

Scanning electron microscopy (SEM) is a crucial technique utilized for the examination of microstructure morphology through the acquisition of high-resolution images. It enables the detection of any structural transitions within the sample. Figure 10 illustrates the morphology of the oil globules that were formed following the emulsification process of the optimized HME S-SNEDDS formulation. The globules in the optimized formulation exhibited predominantly round or spherical morphology and a uniform size distribution, as verified by particle size analysis. The SNEDDS exhibited a consistent globule size in the study.

### 3.13. Dissolution Studies

**Figure 11:**
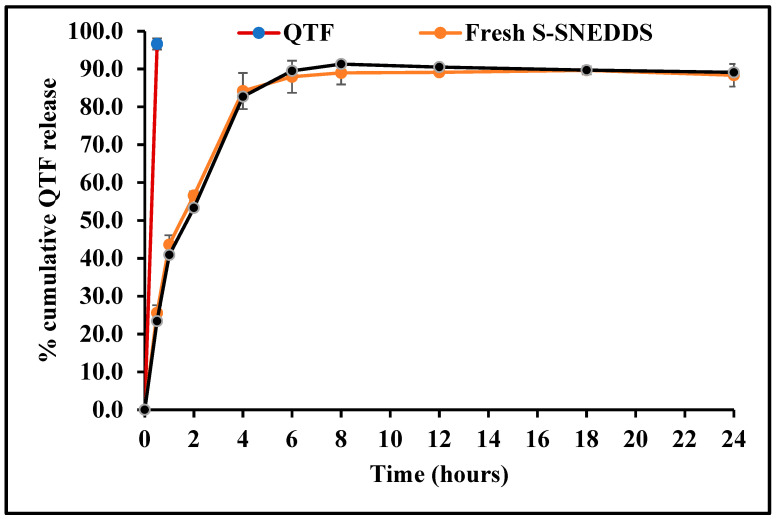
In vitro drug release profiles of quetiapine fumarate (pure API) optimized HME S-SNEDDS (3-month stability at 40 °C/75 ± 5% RH) and optimized HME S-SEDDS (fresh formulation).

The in vitro dissolution profiles of hard gelatin capsules filled with optimized QTF-loaded S-SNEDDS formulation and pure QTF in 0.1 N HCl medium (pH 1.2) at 37 ± 0.5 °C are depicted in Figure 11. It was observed with pure drug that more than 95% QTF was released within 30 min, while only 56.6% drug was released in 120 min from the S-SNEDDS formulation. QTF has a pH-dependent solubility profile, with a higher solubility in the acidic environment. Thus, the dissolution profile was immediate under testing conditions. Upon contact with the dispersion medium, SNEDDS form o/w nanoemulsion. The free energy essential for emulsification is deficient, helping spontaneous emulsification. The first step in drug release is the formation of an interface between the nanoemulsion droplets and the aqueous dissolution medium resulting in the formation of the liquid crystalline phases at the oil globules surface [40]. The entrapment of QTF molecules within the formed nanoemulsion oil globules and slow drug diffusion from the oil phase to the aqueous phase could contribute to the extended-release behavior from the SNEDDS formulation. The release of hydrophobic drugs from this O/W NE type involves two consecutive steps, starting with the partitioning (diffusion) of the loaded hydrophobic drug from the oil phase into surfactant and then into the aqueous dissolution medium, thus extending the drug release from these drug delivery systems [41].

In order to understand the drug release mechanism from the optimized S-SNEDDS formulation, the release data were fitted to four conventional release models using the DDSolver software, as shown in Table 10. The analysis of regression was performed for zero-order (R^2^ = 0.6708), first-order (R^2^ = 0.9801), Higuchi (R^2^ = 0.9787), and Korsmeyer–Peppas (R^2^ = 0.9843) models. Mathematical model fitting revealed that the release profile of the optimized S-SNEDDS formulation followed the Korsmeyer–Peppas model. The calculated slope (n = 0.522) of the Korsmeyer–Peppas model (0.5 < n < 1.0) demonstrated non-Fickian drug release profiles which is controlled by both erosion and diffusion mechanisms.

### 3.14. Stability

The physicochemical stability of the optimized S-SNEDDS formulation was evaluated under accelerated storage conditions over 3 months (last time point tested). The absence of the drug melting peaks in the stability samples of DSC and PXRD demonstrated the preservation of the amorphous nature of the drug, and no significant shift was observed in the melting peaks of recrystallized lipids (Figure 7 and Figure 9). These observations show the stability of the prepared QTF-loaded HME S-SNEDDS. Moreover, the HME extrudates did not show any significant (*p* > 0.05) change in QTF content—98.9 ± 2.3 initial and 97.8 ± 3.1% after 3 months—and drug release rate over the tested storage period (Figure 11).

## 4. Conclusions

The S-SNEDDSs were successfully prepared and optimized for QTF via continuous HME technology. The developed SNEDDS were optimized based on their emulsifying properties and globule size using the response surface methodology. The optimized formulation was evaluated for thermal behavior, crystallinity, surface morphology, physicochemical incompatibility, accelerated stability, and in vitro drug release studies. The optimized formulation showed the desired particle size distribution characteristics and spontaneous emulsification properties. Moreover, the developed S-SNEDDS revealed its amorphous nature, and showed the physicochemical stability for 3 months at 40 °C/75%RH, proving that the appropriate excipients and processing conditions were used. To our knowledge, this is the first investigation that reports the systematic preparation of QTF-loaded S-SNEDDS via a single-step continuous HME technology. In conclusion, this research can guide future investigations upon the implementation of continuous manufacturing processes for S-SNEDDS. Moreover, the developed formulation vehicle could be a potential alternative to the conventional marketed drug product during the therapeutic management of schizophrenia and manic episodes.

## Figures and Tables

**Table 1:** Independent and dependent variables with their coded levels of CCD.

Independent Variables	Coded Levels				
	−α	−1	0	+1	+α
**X_1_; Capmul^®^ MCM (% *w*/*w*)**	7.92893	10	15	20	22.0711
**X_2_; Surfactant–co-surfactant ratio**	1.58579	2.0	3.0	4.0	4.41421
**X_3_; Surfactant type**	Categorical factor (Gelucire^®^ 44/14 and Gelucire^®^ 48/16)				
**Dependent variables**	Y_1_; globule size (nm)				
	Y_1_; emulsification time (min)				

**Table 2:** Composition of hot melt extruded quetiapine fumarate self-nano emulsifying delivery systems as per central composite design.

Formulation	Run	Capmul^®^ MCM (% *w*/*w*, X_1_)	Surfactant Type (X_3_)	Surfactant–Co-Surfactant Ratio (X_2_)	Surfactant (% *w*/*w*)	Propylene Glycol (% *w*/*w*)
**F1**	1	22.0711	Gelucire^®^ 44/14	3	20.946675	6.982225
**F2**	2	15	Gelucire^®^ 44/14	1.58579	21.46449	13.53551
**F3**	3	20	Gelucire^®^ 48/16	2	20	10
**F4**	4	20	Gelucire^®^ 44/14	4	24	6
**F5**	5	7.92893	Gelucire^®^ 48/16	3	31.553302	10.5177675
**F6**	6	15	Gelucire^®^ 44/14	3	26.25	8.75
**F7**	7	10	Gelucire^®^ 48/16	2	26.67	13.33
**F6**	8	15	Gelucire^®^ 44/14	3	26.25	8.75
**F8**	9	7.92893	Gelucire^®^ 44/14	3	31.553302	10.5177675
**F9**	10	20	Gelucire^®^ 44/14	2	20	10
**F10**	11	22.0711	Gelucire^®^ 48/16	3	20.946675	6.982225
**F11**	12	15	Gelucire^®^ 48/16	3	26.25	8.75
**F12**	13	15	Gelucire^®^ 44/14	4.41421	28.53553	6.46447
**F13**	14	10	Gelucire^®^ 44/14	4	32	8
**F14**	15	10	Gelucire^®^ 44/14	2	26.67	13.33
**F11**	16	15	Gelucire^®^ 48/16	3	26.25	8.75
**F15**	17	20	Gelucire^®^ 48/16	4	24	6
**F11**	18	15	Gelucire^®^ 48/16	3	26.25	8.75
**F16**	19	15	Gelucire^®^ 48/16	1.58579	21.46449	13.53551
**F17**	20	10	Gelucire^®^ 48/16	4	32	8
**F6**	21	15	Gelucire^®^ 44/14	3	26.25	8.75
**F18**	22	15	Gelucire^®^ 48/16	4.41421	28.53553	6.46447

All formulations contain QTF (10% *w*/*w*) and Soluplus^®^ and Klucel EF (1:1, 40% *w*/*w*).

**Table 3:** Excipient screening study for QTF in different oils, surfactants, and co-surfactants.

Excipient	Solubility	Excipient	Solubility
Labrafil^®^ M1944 CS	(−)	Glycerol monostearate	(−)
Sesame oil	(−)	Capryol^®^ 90	(−)
Capmul^®^ MCM	(+)	Gelucire^®^ 50/13	(−)
Transcutol^®^ HP	(+)	Kolliphor^®^ RH 40	(−)
Olive oil	(−)	Gelucire^®^ 48/16	(+)
Labrasol^®^	(−)	Kolliphor^®^ HS 15	(−)
Captex^®^ (CCT)	(−)	Cremophor^®^ RH 40	(−)
Castor oil	(−)	Dynasan^®^ 114	(−)
Miglyol^®^ 812N	(−)	Dynasan^®^ 118	(−)
Peceol^®^	(−)	Labrafil^®^ M 2130 CS	(−)
Gelucire^®^ 44/14	(+)	Imwitor^®^ 960 K	(−)
Poloxamer^®^ 188	(−)	Propylene Glycol	(+)
Oleic acid	(−)	Cremophore^®^ EL	(−)

(+) QTF is soluble in the excipients and does not precipitate on cooling; (−): QTF is either soluble in the excipients, but precipitates on cooling or is insoluble in the excipients.

**Table 4:** Design runs of hot melt extruded quetiapine fumarate self-nano emulsifying delivery systems and their responses in terms of globule size and emulsification time (mean ± SD, n = 3).

Formulation	Run	GS (Y_1_, nm)	ET (Y_2_, min)	Formulation	Run	GS (Y_1_, nm)	ET (Y_2_, min)
F1	1	112.4 ± 4.8	21.4 ± 0.1	F11	12	140.3 ± 2.4	17.9 ± 0.2
F2	2	161.1 ± 2.7	36.8 ± 0.2	F12	13	126.8 ± 4.9	17.9 ± 0.1
F3	3	250.6 ± 2.7	49.1 ± 0.5	F13	14	125.7 ± 2.9	16.8 ± 0.1
F4	4	236.3 ± 3.7	17.8 ± 0.2	F14	15	116.4 ± 2.7	34.1 ± 0.4
F5	5	99.8 ± 4.7	14.5 ± 0.1	F11	16	136.2 ± 1.4	17.8 ± 0.2
F6	6	164.7 ± 6.9	19.4 ± 0.3	F15	17	256.7 ± 0.9	34.3 ± 0.3
F7	7	244.2 ± 5.9	17.2 ± 0.1	F11	18	135.4 ± 4.7	17.3 ± 0.1
F6	8	168.9 ± 2.8	19.5 ± 0.1	F16	19	90.2 ± 1.1	25.2 ± 0.2
F8	9	87.1 ± 4.8	23.6 ± 0.1	F17	20	92.1 ± 1.2	3.4 ± 0.05
F9	10	153.9 ± 3.2	30.4 ± 0.3	F6	21	157.6 ± 1.9	19.4 ± 0.3
F10	11	131.3 ± 6.9	59.6 ± 0.7	F18	22	159.5 ± 2.0	4.7 ± 0.04

**Table 5:** Results obtained from ANOVA testing for a quadratic model of CCD for optimization of globule size and emulsification time of QTF-loaded S-SNEDDS.

Source	Particle Size (Y_1_, nm)				Emulsification Time (Y_2_, min)			
	Sum of Squares	Degree of Freedom	F-Value	*p*-Value	Sum of Squares	Degree of Freedom	F-Value	*p*-Value
**Model**	71,923.24	11	1011.38	<0.0001	3556.44	11	1928.94	<0.0001
***X*_1_**	53,981.15	1	8349.85	<0.0001	911.58	1	5438.65	<0.0001
***X*_2_**	6745.55	1	1043.41	<0.0001	815.39	1	4864.74	<0.0001
***X*_3_**	3238.73	1	500.97	<0.0001	4.68	1	27.93	0.0004
***X*_1_ *X*_2_**	225.78	1	34.92	0.0001	1.71	1	10.21	0.0096
***X*_1_ *X*_3_**	9.02	1	1.39	0.2649	1095.48	1	6535.83	<0.0001
***X*_2_ *X*_3_**	243.46	1	37.66	0.0001	0.0579	1	0.3456	0.5696
***X*_1_^2^**	2659.34	1	411.35	<0.0001	353.39	1	2108.4	<0.0001
***X*_2_^2^**	3082.2	1	476.76	<0.0001	18.54	1	110.62	<0.0001
***X*_1_ *X*_2_ *X*_3_**	95.91	1	14.84	0.0032	4.06	1	24.23	0.0006
***X*_1_^2^ *X*_3_**	872.34	1	134.93	<0.0001	189.66	1	1131.55	<0.0001
***X*_2_^2^ *X*_3_**	37.15	1	5.75	0.0375	78.69	1	469.48	<0.0001
**Residuals**	64.65	10			1.68	10		
**Lack of fit**	55.38	6	3.98	0.1010	1.46	6	4.57	0.0815
**Pure error**	9.27	4			0.2133	4		
**Total**	71,987.89	21			3558.11	21		
**Adjusted R^2^**	0.9981				0.9990			
**Predicted R^2^**	0.9942				0.9969			
**Adequate precision**	102.8893				185.8618			

***p*-values** less than 0.05 indicate significant model terms and values greater than 0.1000 indicate the model terms are not significant. Non-significant **lack of fit** is good—we want the model to fit.

**Table 6:** Criteria of variables and responses for the optimization step.

Variable	Goal	Lower Limit	Upper Limit
**Oil concentration (% *w*/*w*)**	Is in range	10	20
**Surfactant–co-surfactant ratio**	Is in range	2.0	4.0
**Surfactant type**	Equal to ⟶ Gelucire^®^ 48/16	------	-----------
**Globule size (nm)**	Minimize	87.1	256.7
**Emulsification time (min)**	Minimize	3.4	59.6

**Table 7:** Results of validation trials of the proposed formulation (Mean, n = 3).

Response	Predicted Value	Results of Validation Trials	95% CI (Low)	95% CI (High)
**Globule size (nm)**	89.099	92.27	84.64	93.55
**Emulsification time (min)**	3.4	3.38	3.23	3.57

**Table 8:** Polydispersity index, zeta potential, and drug content of quetiapine fumarate-loaded HME solid self-nanoemulsifying delivery systems prepared by CCD.

Formulation	Run	PDI	Zeta Potential (mV)	Drug Content (%)
F1	1	0.28 ± 0.10	−13.4 ± 1.7	102.2 ± 1.3
F2	2	0.35± 0.02	−11.0 ± 0.9	94.6 ± 1.0
F3	3	0.62 ± 0.21	−6.8 ± 0.7	101.9 ± 2.2
F4	4	0.29 ± 0.15	−23.0 ±1.8	100.4 ± 1.7
F5	5	0.41 ± 0.03	−19.2 ± 2.9	98.5 ± 1.3
F6	6	0.20 ± 0.09	−18.3 ± 0.9	102.5 ± 0.8
F7	7	0.65 ± 0.19	−9.29 ± 0.8	104.9 ± 1.8
F6	8	0.47 ± 0.02	−14.7 ± 0.6	104.7 ± 1.3
F8	9	0.29 ± 0.06	−14.6 ± 1.2	96.5 ± 2.6
F9	10	0.21 ± 0.04	−6.5 ± 2.2	96.5 ± 5.2
F10	11	0.27 ± 0.04	−8.7 ± 0.5	94.9 ± 1.4
F11	12	0.58 ± 0.17	−18.3 ± 1.9	94.4 ± 6.6
F12	13	0.34 ± 0.03	−12.0 ± 1.5	98.3 ± 3.7
F13	14	0.32 ± 0.03	−12.9 ± 2.3	96.7 ± 0.5
F14	15	0.32 ± 0.02	−13.7 ± 0.4	102.2 ± 7.5
F11	16	0.24 ± 0.09	−9.6 ± 0.1	93.7 ± 0.1
F15	17	0.24 ± 0.03	−14.5 ± 3.8	97.1 ± 2.6
F11	18	0.86 ± 0.14	−11.9 ± 3.4	95.6 ± 1.2
F16	19	0.29 ± 0.04	−18.3 ± 0.8	98.8 ± 3.6
F17	20	0.32 ± 0.02	−17.1 ± 3.4	92.7 ± 2.5
F6	21	0.26 ± 0.07	−11.9 ± 2.5	91.0 ± 2.3
F18	22	0.65 ± 0.08	−17.8 ± 3.0	98.5 ± 7.6

**Table 9:** Content uniformity testing as per USP for quetiapine fumarate loaded SNEDDS capsules.

Capsule #	1	2	3	4	5	6	7	8	9	10
**Assay (%)**	97.5	96.9	98	100.5	97.4	98.8	95.9	98.7	96.6	96.5
**n**	10	**L1**	15.0 unless otherwise specified			**L2**	25.0 unless otherwise specified			
**Average**	97.7	**SD**	1.4	**M**	**98.5**	**Acceptability constant (k)**				**2.4**
**Acceptance value**		**4.1**	**Low side**	**73.9**	**High side**	**123.1**				
**Low side**	[1-(0.01) (L2)] M		**High side**	[1+(0.01) (L2)] M		**Acceptance value**		98.5 -Average + k.SD		
**Case**	**M (case 1) is to be applied when the target assay is ≤101.5**									
**Acceptance** **criteria**	**The acceptance value should be ≤L1**									
	**The assay of each capsule should be between the calculated low and high sides**									

**Table 10:** Model equations for release data fitting for the optimized HME S-SNEDDS.

Model	Equation
Zero-order	M_0_ − M = k·t
First-order	ln M = k·t
Higuchi	M_0_ m = k·t^1/2^
Korsmeyer–Peppas	log (M_0_ − M) = n log t + log k
Here, M_0_ and M represent the initial drug content at the time t_o_ and the drug content remaining at time t, respectively. Zero-order model: % drug released vs. time; First-order model: Amount of drug remaining vs. time; Higuchi model: % drug released vs. square root of time; Korsmeyer-Peppas model: log % drug released vs. log time.	

## Data Availability

The data presented in this study are available within the article.
